# First Isolation of New Canine Parvovirus 2a from Tibetan Mastiff and Global Analysis of the Full-Length *VP2* Gene of Canine Parvoviruses 2 in China

**DOI:** 10.3390/ijms150712166

**Published:** 2014-07-09

**Authors:** Zhijun Zhong, Luqi Liang, Juan Zhao, Xiaoyang Xu, Xuefeng Cao, Xuehan Liu, Ziyao Zhou, Zhihua Ren, Liuhong Shen, Yi Geng, Xiaobin Gu, Guangneng Peng

**Affiliations:** 1 College of Veterinary Medicine, Key Laboratory of Animal Disease and Human Health of Sichuan, Sichuan Agricultural University, Ya’an 625014, Sichuan, China; E-Mails: zhongzhijun488@126.com (Z.Zhong); liangluqi@126.com (L.L.); xiaoyangxv@126.com (X.X.); caoxuefengcxf@126.com (X.C.); liuxuehan1986@126.com (X.L.); zhouziyao988@gmail.com (Z.Zhou); zhihua_ren@126.com (Z.R.); shenlh@sicau.edu.cn (L.S.); gengyisicau@126.com (Y.G.); guxiaobin198225@126.com (X.G.); 2 Animal Nutrition Institute, Sichuan Agricultural University, Ya’an 625014, Sichuan, China; E-Mail: zj308186438@gmail.com

**Keywords:** canine parvovirus, Tibetan mastiff, new CPV-2a, *VP2* gene, phylogenetic analysis

## Abstract

Canine parvovirus 2 (CPV-2) was first identified in 1978, and is responsible for classic parvoviral enteritis. Despite the widespread vaccination of domestic carnivores, CPVs have remained important pathogens of domestic and wild carnivores. In this study, we isolated CPV-2 from Tibetan mastiffs and performed a global analysis of the complete *VP2* gene sequences of CPV-2 strains in China. Six isolates were typed as new CPV-2a, according to key amino acid positions. On a phylogenetic tree, these six sequences formed a distinct clade. Five isolates occurred on the same branch as KF785794 from China and GQ379049 from Thailand; CPV-LS-ZA1 formed a separate subgroup with FJ435347 from China. One hundred ninety-eight sequences from various parts of China and the six sequences isolated here formed seven distinct clusters, indicating the high diversity of CPVs in China. Of 204 *VP2* sequences, 183 (91.04%) encoded the mutation Ser297Ala, regardless of the antigenic type, implying that most Chinese CPV-2 strains contain the *VP2* mutation Ser297Ala. However, the biological significance of this change from prototype CPV-2a/2b to new CPV-2a/2b types remains unclear. This study is the first to isolate new CPV-2a from the Tibetan mastiff. Our data show that new CPV-2a/2b variants are now circulating in China.

## 1. Introduction

Two kinds of parvovirus can infect canines. One is the minute virus of canines (MVC) and the other is canine parvovirus 2 (CPV-2). MVC is also referred to as canine parvovirus 1 (CPV-1) and was isolated from a diseased dog in 1970, when it was initially considered a nonpathogenic virus. In early 1978, a new virus was identified in dogs with hemorrhagic enteritis and leucopenia [1,2]. To differentiate it from CPV-1, the new virus was designated CPV-2. Although CPV-1 and CPV-2 have both been called parvoviruses, they belong to different genera. CPV-1 belongs to the genus *Bocavirus*, subfamily *Parvovirinae*, family *Parvoviridae*, whereas CPV-2 belongs to the genus *Parvovirus*, subfamily *Parvovirinae*, family *Parvoviridae*.

CPV-2 is a small nonenveloped, single-stranded DNA virus (5.2 kb), and is considered to be a highly contagious etiological agent, causing high mortality in young dogs (two prominent clinical forms induce acute hemorrhagic enteritis and myocarditis) [3]. Puppies between 6 weeks and 6 months of age appear to be most susceptible. The infected dogs show acute gastroenteritis, characterized by loss of appetite, vomiting, fever, diarrhea (from mucoid to hemorrhagic), and leucopenia [3]. CPV-2 was first identified in 1978, and causes high morbidity and frequent mortality of up to 10%. In the 1980s, a new CPV-2 strain emerged and was designated CPV-2a. The first known CPV-2a (M24003) strain differs from the prototype CPV-2 at five amino acid positions in *VP2* (one of them, Val555Ile, is unique to this virus) [3,4]. However, recent studies have reported that many CPV-2a strains do not carry the Val-to-Ile substitution at position 555 [5,6,7]. The virus quickly mutated again and a new strain, CPV-2b, emerged in 1984 [8]. CPV-2b differs from CPV-2a in one (Asn426Asp) amino acid. CPV-2a and CPV-2b are still the parvovirus species that most commonly cause disease in canines globally. In 2000, a new mutation, Asp426Glu in *VP2* located on the capsid surface, was first identified in Italy, giving rise to another antigenic variant, designated CPV-2c [9]. This strain was soon reported in many regions, including Europe, South and North America, and Asia [10,11]. In recent years, two antigenic variant strains, new CPV-2a and new CPV-2b, carrying the Ser297Ala mutation as well as the variants of the original CPV-2 (Met87Leu, Ile101Thr, Ala300Gly, and Asp305Tyr), were found circulating in many counties, including China [12,13]. It can be concluded that different antigenic variants of CPV-2 predominate in different countries throughout the world.

In China, hemorrhagic enteritis in dogs caused by CPV-2 was first reported in 1983. In 1986, CPV-2a replaced CPV-2 as the predominant isolate in China [14]. CPV-2b emerged during 1997 and circulated together with CPV-2a, but the isolation frequencies of the two strains appear to differ according to geographic region. The CPV-2a viruses are distributed in all parts of China, whereas CPV-2b only circulates in the southern areas of China [13,14,15]. CPV-2c was first identified in Jilin Province and so far, this has been the only report of CPV-2c in China [16]. In recent years, new CPV-2a and new CPV-2b have been circulating together in China, and new CPV-2a has become the predominant CPV type in China [13,17].

The Tibetan mastiff is a canine species known as the most ferocious dog in the world, and is a domesticated pet in many countries. Tibetan mastiffs have been living on the Qinghai–Tibetan Plateau (over 3000 m above sea level) for more than 1000 years [18]. This species is mainly distributed in Tibet, Qinghai, and Sichuan Provinces in China. Because it has been domesticated as a pet and has thus come into contact with other animals, many pathogens, including H3N2 canine influenza virus, canine distemper virus, and *Toxoplasma gondii* infection, have become potential threats to the health of Tibetan mastiffs [19,20,21]. However, there is little knowledge of CPV-2 infections in the Tibetan mastiff, especially in pups. In this study, six CPV-2 strains were isolated from Tibetan mastiffs with suspected CPV-2 infection in Sichuan Province, China. According to their nucleotide sequences and a phylogenetic analysis, the six isolates all belong to a novel CPV-2a antigenic variant. To our knowledge, this is the first report of CPV-2a isolated from the Tibetan mastiff.

## 2. Results

### 2.1. PCR Amplification of a Unique Fragment of the VP2 Gene and Virus Isolation

**Figure 1:**
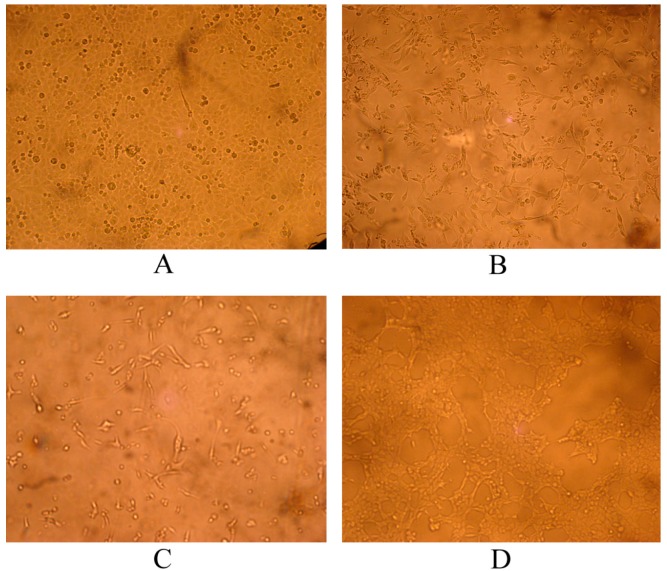
CPV-infected F81 cell line showed cellular changes (×100). (**A**) normal F81 cells; (**B**) tapering cells; (**C**) cytomixis and rounding cells; and (**D**) plaque-forming cells.

**Figure 2:**
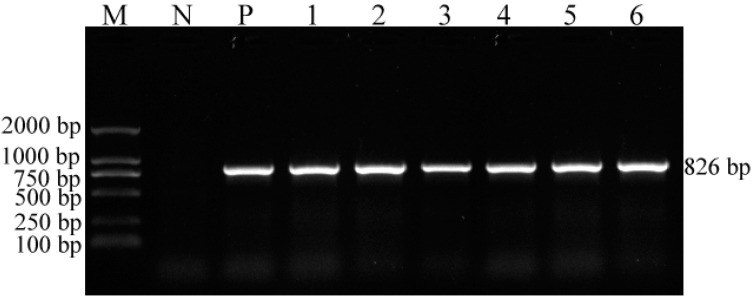
Specific fragment of the *VP2* gene (826 bp) was amplified in six samples with primer 1 and primer 2. Lane **M**, DNA marker (100 bp); lane **N**, blank control; lane **P**, positive control (SCDN strain); lanes **1**–**6**: 826-bp PCR product of CPV-YA-ZA1, CPV-YA-ZA2, CPV-YA-ZA4, CPV-YA-ZA5, CPV-LS-ZA1, and CPV-CD-ZA1.

Seven fecal samples from Tibetan mastiffs showed positive results with the Anigen Rapid CPV Ag Test Kit (Bionote Inc., Seoul, Korea), which were verified by PCR with primers P1 and P2. A specific fragment (826 bp) of the *VP2* gene was amplified from the seven samples (data not shown), confirming the presence of CPV-2 in the samples. Feline kidney 81 cells were inoculated with the seven PCR-positive samples. Cytopathic effects (CPE), including tapering cells, cytomixis, rounding cells, and plaque-forming cells appeared in six cell samples at 6–13 days after inoculation (Figure 1). Of the seven positive samples, six isolates were adapted to F81 cells after three passages. The culture of one of the seven samples (laboratory number: CPV-YA-ZA3) failed. PCR with primers P1 and P2 was also used to detect the *VP2* gene of CPV-2 in the cell cultures. A specific fragment (826 bp) was observed in the six cultured isolates (Figure 2), confirming that CPV-2 was successfully isolated from the six samples.

### 2.2. CPV-2 Characterization

#### 2.2.1. TCID50 Test

The 50% tissue culture infective dose (TCID_50_) was determined for the six isolates. As shown in Table 1, when treated with heat, acid, organic solvent, and 5-iodo-2'-deoxyuridine (5-IDUR), the six isolates were sensitive to 5-IDUR, but were resistant to heat, acid, and organic solvent compared with the blank control. These results are consistent with the characteristics of DNA viruses, and indicate that the six isolated strains are DNA viruses.

**Table 1:** TCID_50_ of isolates and reference strain SCDN (Strain that isolated from Bulldog in Sichuan Province) when treated with heat, acid, organic solvent, or 5-IDUR.

Condition	SCDN TICD_50_/mL	CPV-YA-ZA1 TICD_50_/mL	CPV-YA-ZA2 TICD_50_/mL	CPV-YA-ZA4 TICD_50_/mL	CPV-YA-ZA5 TICD_50_/mL	CPV-CD-ZA1 TICD_50_/mL	CPV-LS-ZA1 TICD_50_/mL
Control	1.6 × 10^5^	1.3 × 10^5^	1.0 × 10^5^	1.6 × 10^5^	1.3 × 10^5^	2.0 × 10^5^	0.8 × 10^5^
60 °C	0.9 × 10^5^	1.0 × 10^5^	0.8 × 10^5^	1.4 × 10^5^	0.9 × 10^5^	1.8 × 10^5^	0.4 × 10^5^
pH 3	0.6 × 10^5^	0.5 × 10^5^	0.3 × 10^5^	0.8 × 10^5^	0.6 × 10^5^	1.3 × 10^5^	0.1 × 10^5^
Ether	0.7 × 10^5^	0.6 × 10^5^	0.4 × 10^5^	0.9 × 10^5^	0.5 × 10^5^	1.2 × 10^5^	0.2 × 10^5^
5-IDUR	0.4 × 10^3^	0.7 × 10^3^	0.6 × 10^3^	0.9 × 10^3^	0.9 × 10^3^	1.9 × 10^3^	0.9 × 10^3^

#### 2.2.2. Viral Hemagglutination (HA)

HA at pH 7.2 and a temperature of 4 °C was used to identify the six isolates. As shown in Table 2, the susceptibility of erythrocytes to the CPVs differed, but the six isolates agglutinated porcine red blood cells (RBCs) strongly. The six isolates also agglutinated feline RBCs, but agglutinated other erythrocytes less sensitively or negligibly. These results are consistent with those of other studies [22,23].

**Table 2:** Hemagglutination spectra of the six isolates and reference strain SCDN.

Strain Name	Pig	Cow	Sheep	Mouse	Dog	Cat	Chicken
SCDN	2^9^	<2^1^	<2^1^	<2^1^	<2^1^	2^3^	<2^1^
CPV-YA-ZA1	2^11^	<2^1^	<2^1^	<2^1^	<2^1^	2^4^	<2^1^
CPV-YA-ZA2	2^9^	<2^1^	<2^1^	<2^1^	<2^1^	2^3^	<2^1^
CPV-YA-ZA4	2^9^	<2^1^	<2^1^	<2^1^	<2^1^	2^2^	<2^1^
CPV-YA-ZA5	2^10^	<2^1^	<2^1^	<2^1^	<2^1^	2^3^	<2^1^
CPV-CD-ZA1	2^11^	<2^1^	<2^1^	<2^1^	<2^1^	2^4^	<2^1^
CPV-LS-ZA1	2^7^	<2^1^	<2^1^	<2^1^	<2^1^	2^1^	<2^1^

### 2.3. PCR Amplification of the Complete VP2 Gene and Its Sequence

**Figure 3:**
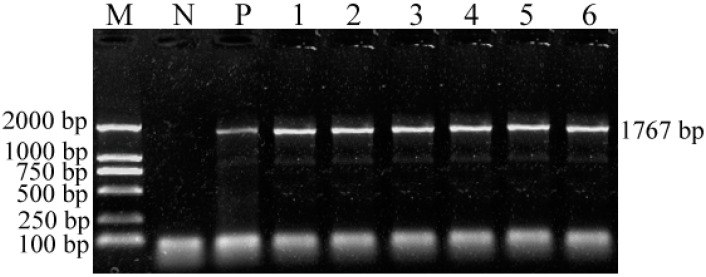
Complete *VP2* genes, obtained from the six isolates using primer 3 and primer 4, were confirmed in the recombinant plasmids. Lane **M**, DNA marker (100 bp); lane **N**, negative control; lane **P**, positive control (SCDN strain); lanes **1**–**6**: 1767-bp complete *VP2* gene sequences of CPV-YA-ZA1, CPV-YA-ZA2, CPV-YA-ZA4, CPV-YA-ZA5, CPV-LS-ZA1, and CPV-CD-ZA1, respectively, in the recombinant plasmids

**Figure 4:**
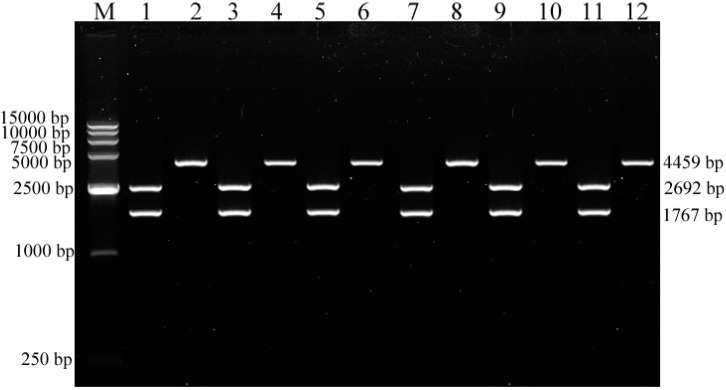
Doubly digested (*Hin*dIII and *Eco*RI) 1767-bp complete *VP2* genes inserted into recombinant plasmids. Lane **M**, DNA marker (250 bp); lanes **1**, **3**, **5**, **7**, **9**, and **11**: recombinant plasmid DNA of CPV-YA-ZA1, CPV-YA-ZA2, CPV-YA-ZA4, CPV-YA-ZA5, CPV-LS-ZA1, and CPV-CD-ZA1, respectively, digested with *Hin*dIII and *Eco*RI; lanes **2**, **4**, **6**, **8**, **10**, and **12**: recombinant plasmid DNA of CPV-YA-ZA1, CPV-YA-ZA2, CPV-YA-ZA4, CPV-YA-ZA5, CPV-LS-ZA1, and CPV-CD-ZA1, respectively digested with *Hin*dIII

PCR products of 1767 bp were observed for the six isolates on agarose gel, indicating that the complete *VP2* gene of CPV-2 was amplified with primers P3 and P4 (data not shown). The purified PCR products were cloned into the pMD^®^ 19-T vector and the presence of the desired insert (1767 bp) in the recombinant plasmid DNA was confirmed by PCR amplification (primers P3 and P4; Figure 3) and double digestion with enzymes *Hin*dIII and *Eco*RI (Figure 4). The correct insertion of theTibetan mastiff *VP2* genes in six different recombinant plasmids was confirmed and the positive recombinant plasmids were sequenced by Invitrogen™ of Shanghai Biotechnology Co., Ltd. (Shanghai, China). When the restriction enzyme site sequences are disregarded, the actual length of the sequence is 1755 bp. The sequences were deposited in GenBank under accession numbers HQ651237 for CPV-YA-ZA1, JQ996151 for CPV-YA-ZA2, JQ996152 for CPV-YA-ZA4, JQ996153 for CPV-YA-ZA5, JQ996154 for CPV-CD-ZA1, and JQ996155 for CPV-LS-ZA1.

### 2.4. Phylogenetic Tree and Amino Acid Analysis

**Figure 5:**
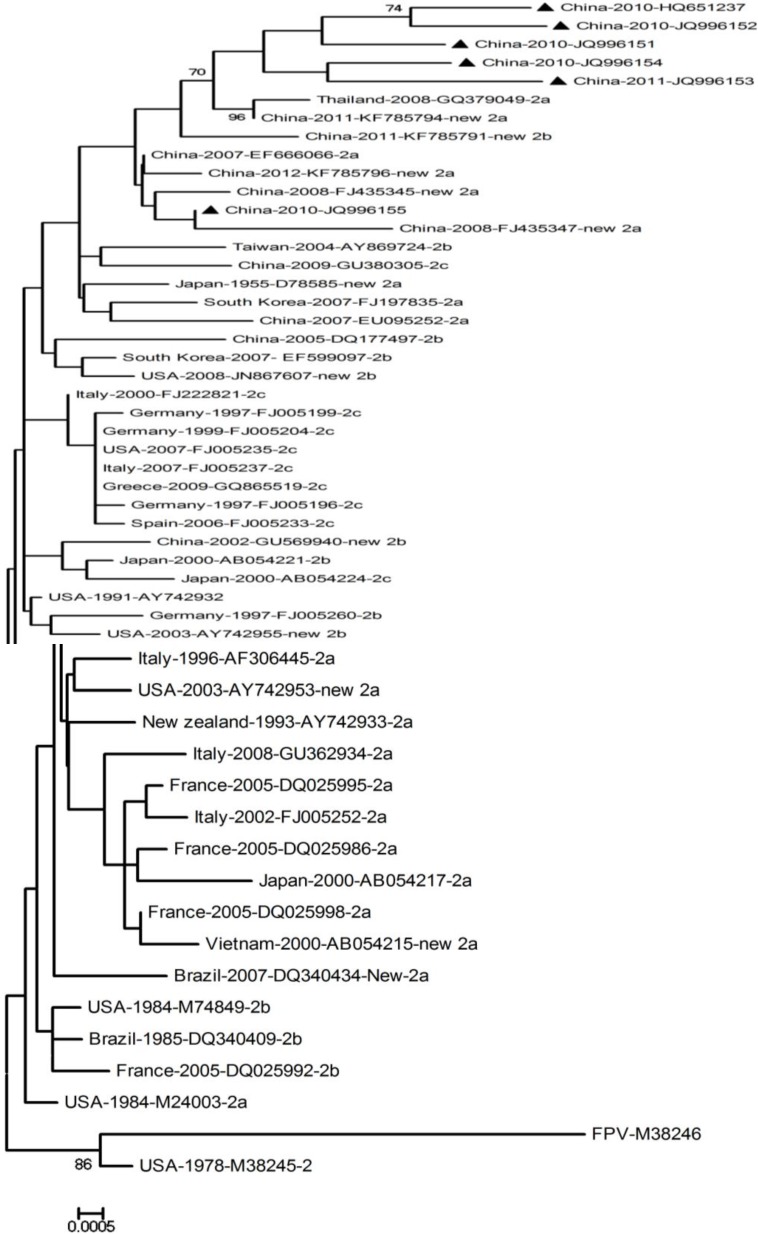
Phylogenetic tree of six isolates and parvovirus reference strains based on the complete nucleic acid sequences of their *VP2* genes. The analysis was performed with the NJ method based on 1000 replicates using the MEGA 4.0 software, and bootstrap values >70% are shown at the corresponding node. Following the names of the countries are the years, GenBank accession numbers, and the types of sequence.

The six sequences identified in this study, 46 published full sequences of the CPV *VP2* gene, and one FPV *VP2* gene from GenBank were analyzed. The CPV reference strains CPV-2, CPV-2a, CPV-2b, CPV-2c, new CPV-2a, and new CPV-2b were collected from various parts of the world, including Europe, America, and Asia. A neighbor-joining (NJ) phylogenetic tree was constructed with the MEGA version 4.0 software, with the Kimura two-parameter model (Figure 5). The phylogenetic tree shows that the six sequences isolated in this study predominantly cluster in a distinct clade. Five isolates are located on the same branch as KF785794 from China and GQ379049 from Thailand, whereas CPV-LS-ZA1 forms a subgroup with FJ435347 from China. The six sequences all cluster within the Asian strain clade, so they share the same ancestral origin.

**Figure 6:**
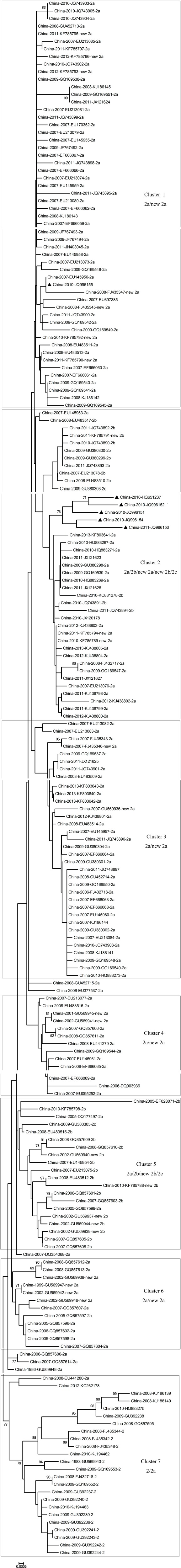
Global analysis of 198 full-length CPV-2 *VP2* genes (1755 bp) isolated in China and retrieved from GenBank, together with the six sequences isolated in this study. The analysis was performed with the NJ method based on 1000 replicates using the MEGA 4.0 software, and bootstrap values >70% are indicated at the corresponding nodes. Following the names of the countries are the years, GenBank accession numbers, and the types of sequence.

To analyze all the CPV-2 *VP2* sequences from China deposited in GenBank, 198 sequences of the full-length *VP2* gene (1755 bp) were extracted, including 15 CPV-2, 115 CPV-2a, 24 CPV-2b, two CPV-2c, 17 new CPV-2a, six new CPV-2b, and 19 uncharacterized strains. CPV-2a (115, 56.17%) is the predominant type of CPV-2 in China, followed by the CPV-2b (24, 11.76%). As shown in Figure 6, the six sequences isolated in this study and the other 198 sequences from China deposited in the GenBank database formed seven distinct clusters (clusters 1–7). Clusters 2 and 5 include more than two types of parvoviruses, indicating strong variation within the two clusters. CPV-2a and new CPV-2a strains are found in clusters 1, 3, 4, and 6. Five of the six sequences in this study occurred in cluster 2, whereas the JQ996155 sequence did not. Moreover, only two CPV-2c sequences were collected in our study, which indicates that CPV-2c is seldom isolated in China. We also found that cluster 1 (54 strains) and cluster 2 (41 strains) contain the most strains, indicating that these two groups are the major clusters occurring in China. We also found that more CPV-2 sequences were submitted to GenBank in 2007 (47 strains) and 2009 (38 strains) than in other years, which implies that CPV-2 infections were very serious in China during that period.

We also analyzed the amino acid residues encoded by the *VP2* gene in the six isolates. The results are shown in Table 3. The deduced amino acid residues of *VP2* for the isolates were compared with those of the reference strains at critical positions in the CPV-2 *VP2* protein. The six isolates were typed as new CPV-2a according to the key amino acid residues at positions 87, 101, 297, 300, 305, 426, and 555. Our results indicate that the six isolates from the Tibetan mastiff belong to antigenic type CPV-2a. Interestingly, the Ser297Ala mutation occurs in all six isolates. We also analyzed this mutation in the 198 Chinese sequences from GenBank, 177 (89.39%) of which displayed the Ser297Ala mutation. Moreover, the residues at position 324 in our six isolates were all altered. HQ651237, JQ996151, JQ996154, and JQ996155 displayed the mutation Tyr324Ile; the Tyr324Asn mutation was detected in JQ996152; and the Tyr324Phe mutation in JQ996153. The mutation Tyr324Ile was observed in 69 (34.85%) *VP2* sequences of the 198 Chinese CPV-2 *VP2* sequences in GenBank. Although this mutation does not result in antigenic variation, the substitution at residue 324 is adjacent to residue 323, which is considered to affect the infection of canine cells. Some strains have recently been reported to have the mutation Gly300Asp, but no sequence from China has this mutation.

**Table 3:** Amino acid variations in the *VP2* proteins of the six CPVs.

Strain	Amino Acid Position													
	80	87	93	101	103	297	300	305	323	324	426	555	564	568
FPV	Lys	Met	Lys	Ile	Val	Ser	Ala	Asp	Asp	Tyr	Asn	Val	Asn	Ala
CPV2	Arg	Met	Asn	Ile	Ala	Ser	Ala	Asp	Asn	Tyr	Asn	Val	Ser	Gly
CPV2a	Arg	Leu	Asn	Thr	Ala	Ser	Gly	Tyr	Asn	Tyr	Asn	Ile	Ser	Gly
CPV2b	Arg	Leu	Asn	Thr	Ala	Ser	Gly	Tyr	Asn	Tyr	Asp	Val	Ser	Gly
CPV2c	Arg	Leu	Asn	Thr	Ala	Ala	Gly	Tyr	Asn	Tyr	Glu	Val	Ser	Gly
new CPV 2a	Arg	Leu	Asn	Thr	Ala	Ala	Gly	Tyr	Asn	Tyr	Asn	Val	Ser	Gly
new CPV 2b	Arg	Leu	Asn	Thr	Ala	Ala	Gly	Tyr	Asn	Tyr	Asn	Val	Ser	Gly
YAZA1	Arg	Leu	Asn	Thr	Ala	Ala	Gly	Tyr	Asn	Ile	Asn	Val	Ser	Gly
YAZA2	Arg	Leu	Asn	Thr	Ala	Ala	Gly	Tyr	Asn	Ile	Asn	Val	Ser	Gly
YAZA4	Arg	Leu	Asn	Thr	Ala	Ala	Gly	Tyr	Asn	Asn	Asn	Val	Ser	Gly
YAZA5	Arg	Leu	Asn	Thr	Ala	Ala	Gly	Tyr	Asn	Phe	Asn	Val	Ser	Gly
CDZA1	Arg	Leu	Asn	Thr	Ala	Ala	Gly	Tyr	Asn	Ile	Asn	Val	Ser	Gly
LSZA1	Arg	Leu	Asn	Thr	Ala	Ala	Gly	Tyr	Asn	Ile	Asn	Val	Ser	Gly

### 2.5. Homology of the Six Isolates from the Tibetan Mastiff

Compared with 10 references strains from China and other countries (Table 4), the six isolates from the Tibetan mastiff showed nucleotide identities of 98.6%–99.6% and divergences of 0.4%–1.4%. The strain most similar to the reference strains was HQ883267 from Beijing, China, with homologies of 99.0%–99.6%.

**Table 4:** *VP2* gene homology analysis for the six isolates and 10 reference strains.

Accession Number	1	2	3	4	5	6	7	8	9	10	11	12	13	14	15	16
**1**		99.2	99.0	99.4	98.7	99.3	98.5	99.0	99.6	99.0	99.0	98.8	99.0	99.0	98.8	98.9
**2**	0.8		99.0	99.2	99.1	99.0	99.0	99.6	99.6	99.5	99.3	99.4	99.5	99.5	99.4	99.5
**3**	1.0	1.0		99.5	99.4	98.9	98.3	98.9	99.4	98.8	98.8	98.6	98.9	98.8	98.6	98.7
**4**	0.6	0.8	0.5		99.0	99.1	98.5	99.0	99.5	99.0	99.0	98.8	99.0	99.0	98.8	98.9
**5**	1.3	0.9	0.6	1.0		98.9	98.3	99.0	99.0	98.9	98.7	98.7	98.8	98.9	98.7	98.8
**6**	0.7	1.0	1.1	0.9	1.1		98.3	99.0	99.1	98.8	98.7	98.6	98.9	98.8	98.6	98.7
**7**	1.6	1.0	1.7	1.6	1.7	1.7		99.3	98.9	99.3	99.1	99.1	99.1	99.3	99.1	99.2
**8**	1.0	0.4	1.1	1.0	1.0	1.0	0.7		99.4	99.7	99.6	99.5	99.5	99.7	99.5	99.7
**9**	0.4	0.4	0.6	0.5	1.0	0.9	1.1	0.6		99.4	99.4	99.2	99.4	99.4	99.2	99.3
**10**	1.0	0.5	1.2	1.0	1.1	1.2	0.7	0.3	0.6		99.5	99.6	99.6	99.8	99.6	99.7
**11**	1.0	0.7	1.2	1.0	1.3	1.3	0.9	0.4	0.6	0.5		99.5	99.5	99.7	99.5	99.6
**12**	1.2	0.6	1.4	1.2	1.3	1.4	0.9	0.5	0.8	0.4	0.5		99.5	99.7	99.5	99.7
**13**	1.0	0.5	1.1	1.0	1.2	1.1	0.9	0.5	0.6	0.4	0.5	0.5		99.7	99.5	99.7
**14**	1.0	0.5	1.2	1.0	1.1	1.2	0.7	0.3	0.6	0.2	0.3	0.3	0.3		99.8	99.9
**15**	1.2	0.6	1.4	1.2	1.3	1.4	0.9	0.5	0.8	0.4	0.5	0.5	0.5	0.2		99.9
**16**	1.1	0.5	1.3	1.1	1.2	1.3	0.8	0.3	0.7	0.3	0.4	0.3	0.3	0.1	0.1	

The upper right values are the percentage identities of the *VP2* gene among viruses and the lower left values are the divergences; **1**–**6**: CPV isolates from the Tibetan mastiff (CPV-CD-ZA1, CPV-LS-ZA1, CPV-YA-ZA1, CPV-YA-ZA2, CPV-YA-ZA4, CPV-YA-ZA5, respectively); **7**–**16**: CPV isolates from China and other countries with GenBank accession numbers: GU569943, GU569947, HQ883267, FJ005259, GU569944, FJ222823, AY742951, FJ222821, FJ005214, GQ865519, respectively.

## 3. Discussion

In this study, six strains of new CPV-2a were isolated from young Tibetan mastiff dogs. According their clinical histories, seven dogs in this study were incompletely vaccinated puppies (vaccinated once or twice). Their infection with canine parvovirus may be attributable to either infection before vaccination or the failure of vaccination. Differences in virus types between field viruses and the vaccine virus could be another important reason for some immunization failure in dogs and wildlife. The vaccine available in China is a type CPV-2 vaccine (such as Nobivac Puppy DP and Nobivac DHPPi). The effectiveness of this vaccine against the new CPV-2a virus must be evaluated, because it confers lower and shorter immunity against heterologous CPVs [13,24].

In China, the predominant CPV-2 strains are CPV-2a and CPV-2b [13,14,15,17]. Our results also showed that the predominant types of CPV-2 are CPV-2a (115, 56.17%) and CPV-2b (24, 11.76%) among 204 sequences analyzed in this study. Phylogenetic analysis indicates that the six isolates in our study predominantly cluster in a distinct clade (Figure 5), which might be attributed to the process of local adaptation (six samples were all collected in and around Chengdu, Sichuan, China) as indicated by previous study [13,25].

The Ser297Ala amino acid change in *VP2* is considered the defining mark of the new CPV-2a and new CPV-2b strains [13,26]. However, the biological significance of this mutation for the new CPV-2a/2b types remains unclear. This mutation was present in our six isolates. When we analyzed this mutation in the 198 sequences from China in GenBank, 177 (89.39%) of them displayed the mutation Ser297Ala. The Ser297Ala change appeared in 111 of the 115 CPV-2a strains and in all 24 CPV-2b strains. This result indicates that many CPV-2a/b stains isolated in China can be classified as new CPV-2a or new CPV-2b. According to this criterion, new CPV-2a or new CPV-2b has been the dominant CPV-2 in China for a long time, which is consistent with the results of other studies [13,17].

Previous studies have reported that a distinct mutation, Tyr324Ile, frequently occurs in the *VP2* gene of CPV-2 from Asian, South American and European countries, including China [13,17], Korea [12,27], Thailand [28], Japan [29], India [30,31], Taiwan [32], Uruguay [33] and Hungary [34]. Phylogenetic analysis indicated that the Uruguayan CPV-2a strain is related to CPV-2a strains from China collected during 2006–2009 [33]. Recently, the Tyr324Ile alteration was also detected in Hungarian strains and possessed a “Hungarian-specific” substitution (Ala516Thr) [34]. This is the first time the Tyr324Ile mutation was detected in Europe. In our study, HQ651237, JQ996151, JQ996154, and JQ996155 contained the mutation Tyr324Ile, whereas JQ996152 and JQ996153 had the mutations Tyr324Asn and Tyr324Phe, respectively. To our knowledge, Try324Phe is a unique and previously unrecorded mutation of CPV-2a. Whether the Try324Phe mutation is a new evolutionary event needs more samples isolated from Tibetan mastiffs to verify. Further analysis showed that the Tyr324Ile mutation occurs in 69 (34.85%) of the 198 CPV-2 *VP2* sequences from China in GenBank. Previous studies have shown that the substitution at residue 324 is adjacent to residue 323, which is thought to affect the parvovirus host range, although this mutation does not alter the antigenic properties of the virus [35]. In our study, three CPV-2 *VP2* sequences from the red panda (DG354068), blue fox (GQ857595), and raccoon dog (GQ857614) were also included in the analysis [36,37]. When residues 323 and 324 were examined, the Asn323Asp mutation was only detected in GQ857595. No mutation was detected at residues 324 in DQ354068, GQ857595, or GQ857614. Therefore, further study of more extensive CPV-2 sequences from various animals is required to clarify whether residue 323 affects the parvovirus host range. On a phylogenetic tree, DQ354068 formed a separate branch, indicating its great genetic distance from the other strains. The phylogenetic relationship between GQ857614 and GQ857600 is close, so they may share an ancestral origin. GQ857595 was placed in cluster 7, which contained other CPV-2 strains, indicating that GQ857595 is similar to CPV-2. This result demonstrates that the CPV-2 strains in wild animals are constantly evolving to extend their host ranges. Another study has also shown that evolutionary events have occurred in CPV-2 to extend its host range, such as the mutation of residue 370 in *VP2* detected in the CPV-2 strain infecting the giant panda [36].

## 4. Materials and Methods

### 4.1. Sample Collection and Preprocessing

In 2010–2011, seven rectal swab specimens from Tibetan mastiffs (Table 5) with suspected CPV-2 infection were collected from the Teaching Veterinary Hospital of Sichuan Agricultural University and pet clinics in and around Chengdu, the capital city of Sichuan Province, China. The diagnostic criteria for CPV-2 infection were based on clinical signs, the examination of blood, and a positive result on the Anigen Rapid CPV Ag test kit (Bionote Inc., Gyeonggi-do, Korea). The swabs were washed 3 times in 1 mL of phosphate-buffered saline (PBS, pH 7.2) and then clarified at 12,000 rpm for 10 min at 4 °C. The supernatants were passed through 0.22 μm filters (Millipore, Billerica, MA, USA) and then used for PCR amplification.

**Table 5:** Breed, age, sex, and vaccination status of the seven dogs infected with CPV-2.

Strain Number	Breed	Age	Gender	Vaccination Status	NCBI Accession Numbers
CPV-YA-ZA1	Tibetan mastiff	Two months	Female	Once	HQ651237
CPV-YA-ZA2	Tibetan mastiff	One month	Female	Once	JQ996151
CPV-YA-ZA3	Tibetan mastiff	Three months	Female	Twice	/
CPV-YA-ZA4	Tibetan mastiff	Two months	Female	Once	JQ996152
CPV-YA-ZA5	Tibetan mastiff	Two months	Female	Twice	JQ996153
CPV-LS-ZA1	Tibetan mastiff	Two months	Male	Once	JQ996155
CPV-CD-ZA1	Tibetan mastiff	Three months	Female	Twice	JQ996154

### 4.2. DNA Extraction and VP2 Gene Amplification

An aliquot (100 µL) of the supernatant was used to extract the viral DNA with a Qiagen Viral DNA kit (Qiagen, Hilden, Germany), according to the manufacturer’s instructions [14]. Primers 1 and 2 (P1: 5'-AACGGATGGGTGGAAATCAC-3'; P2: 5'-TAATAGTAGCTTCAGTAATA-3') were used to amplify a unique 826-bp fragment of the *VP2* gene. The PCR reaction was performed in a final volume of 25 μL containing 1.0 μL of viral DNA, 0.125 μL of Ex *Taq* DNA polymerase (5 U/μL), 2.5 μL of 10× Ex *Taq* buffer (Mg^2+^ plus), 2 μL of dNTP mixture (2.5 mmol/L), 1 μL of P1, and 1 μL of P2, made up to 25 μL with sterile distilled water. The standardized PCR protocol for primers 1 and 2 in a 25 μL reaction mixture was incubation at 96 °C for 3 min, followed by 30 cycles of denaturation at 94 °C for 30 s, primer annealing at 52 °C for 35 s, and extension at 72 °C for 3 min; with a final extension at 72 °C for 10 min. The PCR products were analyzed by electrophoresis on 1.5% agarose gels stained with ethidium bromide.

### 4.3. Virus Isolation and Characterization by HA and TCID_50_

The seven PCR-positive samples were used for virus isolation, as described by Nandi, S. [38]. The samples were homogenized in PBS (pH 7.2) and then clarified at 12,000 rpm for 10 min. The supernatants were filtered through 0.22 µm filters (Millipore). The filtrates were then treated with penicillin and streptomycin overnight at 4 °C and used to inoculate feline kidney 81 cells cultured in Dulbecco’s modified Eagle’s medium (Sigma, Saint Louis, MO, USA) with 15% fetal calf serum (Gibco, Logan, UT, USA). The infected monolayers displaying CPE were harvested 4–5 days after infection with three cycles of alternative freezing and thawing. The virus-containing supernatants were used for DNA isolation or stored at –20 °C until further analysis. An HA test and TCID_50_ test were used to characterize the viral isolates [22,23] *.* SCDN (Strain that isolated from Bulldog in Sichuan province) (CPV-2a) strain used as positive control [39].

### 4.4. Six Viral Isolates Identified with PCR

Viral DNA was extracted from the harvested supernatants of the cultures. Primers 3 and 4 (P3, P4) were designed to amplify the complete *VP2* gene. *Hin*dIII: P3: 5'-*AAGCTT*ATGAGTGATGGAGCAGTTCAACCAGAC-3', *Eco*RI: P4: 5'-*GAATTC*TTAATATAATTTTCTAGGTGCTAGTTGA-3'.

The forward primer (P3) and reverse primer (P4) were tagged the restriction site for *Hin*dIII and *Eco*RI, respectively, to allow the complete *VP2* gene of the isolates to be cloned into the pMD^®^ 19-T vector (Takara Biotechnology (Dalian) Co., Ltd., Dalian, China). The 50 µL PCR reactions contained: 2.5 µL of viral DNA, 0.25 µL of Ex *Taq* DNA polymerase (5 U/µL), 5.0 µL of 10× Ex *Taq* buffer (Mg^2+^ plus), 4 µL of dNTP mixture (2.5 mmol/L), 2 µL of P3, and 2 µL of P4, made up to 50 µL with sterile distilled water. The standardized PCR protocol for primers 3 and 4 in a 50 µL reaction mixture was incubation at 96 °C for 3 min, followed by 35 cycles of denaturation at 95 °C for 30 s, annealing at 50 °C for 30 s, and extension at 72 °C for 140 s; with a final extension at 72 °C for 10 min. The PCR products were analyzed by electrophoresis on 1.5% agarose gels stained with ethidium bromide. The PCR-amplified products (1767 bp) were excised from the gel and purified with the QIAquick Gel Extraction kit (Qiagen, Hilden, Germany).

The purified PCR products of 1767 bp were cloned into the bacterial vector pMD^®^ 19-T (Takara Biotechnology (Dalian) Co., Ltd., Dalian, China), according to the manufacturer’s instructions. Positive colonies were confirmed by PCR amplification and double enzymatic digestion.

### 4.5. Nucleotide Sequencing and Analysis

The positive clones were sequenced at the Sequencing Facility, Invitrogen. Neighbor-joining phylogenetic trees were constructed with the MEGA version 4.0 software (Biodesign Institute, Tempe, AZ, USA) with the Kimura two-parameter method [13]. The data analyzed were the nucleotide sequences of the *VP2* genes of the canine parvovirus 2 (CPV-2) isolates from the Tibetan mastiff and another 198 full-length CPV *VP2* genes isolated in China (1755 bp) extracted from GenBank (years 1983–2013). The deduced amino acid sequences of the six isolates were compared with the reference strains at some key *VP2* residues. Sequence alignments were performed with the DNAMAN version 6.0 software. The percentage similarity and divergence of the *VP2* genes among the six isolates and 10 references strains (GU569943, GU569947, HQ883267, FJ005259, GU569944, FJ222823, AY742951, FJ222821, FJ005214, and GQ865519) from China and other countries were analyzed with MegAlign of the DNAStar software (DNASTAR Inc., Madison, WI, USA).

## 5. Conclusions

We isolated CPV-2 from young Tibetan mastiff dogs in China. Six isolates were typed as new CPV-2a based on the key amino acids at positions 87, 101, 297, 300, 305, 426, and 555. A phylogenetic tree showed that our six sequences formed a distinct clade. Five isolates occurred on the same branch as KF785794 from China and GQ379049 from Thailand. CPV-LS-ZA1 formed a separate subgroup with FJ435347 from China. The six sequences identified in this study and 198 sequences from China deposited in GenBank formed seven distinct clusters, which indicates the high level of diversity in the CPVs in China. Of 198 sequences, 177 (89.39%) displayed the mutation Ser297Ala. The incorporation of field strains in the commercial vaccine may allow the effective control of CPV-2 infection in the Tibetan mastiff.
